# Promoting adolescent mental health in Tanzania and Vietnam through a co-created universal school-based initiative: Findings from a mixed method study

**DOI:** 10.1017/gmh.2025.10100

**Published:** 2025-12-01

**Authors:** Fiona Samuels, Emma Samman, Jose Manuel Roche, Carmen Leon-Himmelstine, Edward Amani, Ha Ho, Arnaldo Pellini, Phuong Nguyen, Dayani Mbowe, Georgia Plank, Johnson Mshiu, Ngoc Nguyen, Van Vu, Dao Kieu, Ha Le, Esther Kyungu, Hoang-Minh Dang

**Affiliations:** 1 Wolfson Institute of Population Health, Faculty of Medicine and Dentistry, Queen Mary University of London, UK; 2 ODI, UK; 3 Independent Consultant, UK; 4 Tanzania Training Centre for International Health, United Republic of Tanzania; 5 College of Education, Vietnam National University, Vietnam; 6 Capability Oy, Finland; 7 Camara Education Tanzania, United Republic of Tanzania; 8 National Institute of Medical Research (NIMR), Dar Es Salaam, Tanzania, United Republic of Tanzania; 9 Vietnam National University of Engineering and Technology, Hanoi, Vietnam

**Keywords:** adolescents, mental health promotion, Tanzania, Vietnam, co-creation, universal

## Abstract

This article assesses a 10-month co-created universal school-based mental health (SBMH) promotion initiative for adolescents (10–19). The study combined quantitative and qualitative components. Pre- and post-intervention surveys were conducted in four schools in Tanzania (*n* = 400 baseline, 488 endline, with 100 intervention participants at both) and eight schools in Vietnam (*n* = 1,036 baseline, 893 endline and 436 in panel). In each country, ~90 qualitative interactions (interviews and focus groups) were held at baseline and endline with adolescents, parents, teachers and service providers (total = ~180). In Tanzania, multivariate analysis indicated significant gains among intervention participants relative to peers. Emotional literacy rose 9.5% (*p* = 0.007; *d* = 0.57). Attitudes toward help-seeking (*p* = 0.021; *d* = 0.50) and prosocial behaviors (*p* = 0.043, *d* = 0.38) also improved Active coping increased 15.6% (*p* = 0.006; *d* = 0.55). In Vietnam, emotional literacy increased 5.3% (*p* = 0.012, *η*^2^ = .019), and positively, emotion-focused coping declined 14.4% (*p* = 0.032, *η*^2^ = .015). Qualitative evidence reinforces these findings, and suggested spillover effects for nonparticipants. Overall results indicate that co-created universal SBMH initiatives can improve adolescent well-being and offer viable alternatives to limited adolescent-focused mental health services in LMICs.

## Impact statement

Extensive evidence highlights adolescence as a period of turbulence during which mental ill-health often starts. It also finds that addressing youth mental ill-health early can promote future well-being, with long-lasting effects. While there is growing evidence of what works in high-income countries to address youth mental health, we know less about how to intervene effectively in low- and middle-income countries (LMICs). Relatedly, school-based mental health (SBMH) promotion initiatives have been shown to be effective in many contexts; however, further proof is needed of their effectiveness in LMICs. Our study presents results from piloting an SBMH initiative in Tanzania and Vietnam, co-created with youth using mixed-methods approaches; to our knowledge, this is the first co-created SBMH initiative delivered in either country. Our mixed-methods analysis shows that the initiative positively affected the mental health status of adolescents, literacy and coping strategies and had wider spillover effects on nonparticipants by increasing their mental health awareness. We identify three main areas in which our findings could have an impact and affect practice: first, our findings show that it is possible to co-create an effective intervention with adolescents using schools as entry points in Tanzania and Vietnam, and therefore, that this model could be replicated, adapted appropriately, in other LMICs; second, our study finds that mental health practitioners should work closely with end-users/adolescents when designing and delivering programming as they are often the best placed to know what works best for them; finally, our findings suggest that mental health practitioners should consider hybrid digital and in-person components when designing interventions – the former motivated engagement and protected anonymity, while the latter cultivated trust and connection.

## Background

Mental ill-health affected 970 million people globally in 2019 (WHO, 2022), with the coronavirus disease 2019 (COVID-19) pandemic and cost-of-living crisis accelerating these numbers. Common mental health difficulties, such as anxiety and depression, powerfully predict suicide and longer-term health problems, and cost the global economy US$1 trillion annually (The Lancet Global Health, 2020). Low- and middle-income countries (LMICs) especially struggle with these costs. As anxiety and depression often start during adolescence (Kessler et al., 2007; Patton et al., 2016; WHO, 2021, 2024), targeting interventions to youth may mitigate long-term personal and economic costs while taking advantage of a developmental window of opportunity (Crone and Dahl, 2012).

A large and growing scholarship addresses school-based mental health (SBMH) interventions (Das et al., 2016; Dray et al., 2017; Kern et al., 2017; O’Reilly et al., 2018; Higgen et al., 2022). This literature highlights schools as critical entry points for addressing mental health, the importance of working with school staff alongside psychiatrists and other mental health providers and the need to increase the evidence base regarding what kinds of interventions – promotion/prevention, whole school/universal (Glazzard, 2019) or targeted – work best. However, most studies and related interventions have been carried out in high-income countries (Caldwell et al., 2019), and while a few school-based trials target adolescents with recognized mental health problems in LMICs, none focus on universal interventions (Michelson et al., 2020; Osborn et al., 2021).

An expanding body of literature also explores the benefit of co-created approaches to develop mental health interventions with young people (Chinsen et al., 2025). At their core – and grounded in a long history of participatory research – is the principle that end-users and those with lived experience are central and equal partners in any design process. Consequently, by redistributing power and decision-making to end-users, interventions are likely to become more relevant and effective and achieve higher uptake (Prebeg et al., 2023). Criticisms include unclear definitions and guidance and concerns regarding quality, ethics and the potential co-optation of end-users (Moll et al., 2020; Vargas et al., 2022). However, co-creation with youth in mental health programming remains limited in LMICs. For instance, in their systematic review of 41 studies, Chinsen et al. (2025) identified only one from Africa and two from Asia.

This study contributes to the evidence base by reporting on a 3-year study of a universal school-based Adolescent Mental Health Promotion (AMP) co-created initiative in Tanzania and Vietnam. As far as we are aware, at the time of design and implementation, these were the first co-created universal SBMH initiatives in both countries, that is, they did not explicitly target adolescents with recognized mental health difficulties, instead focused on promoting psychological well-being (see Supplementary Materials [SM5] for details of other SBMH initiatives identified after completion of our study).

Tanzania and Vietnam represent diverse LMIC contexts, differing in economic and technological development, health systems and mental health service provision. These differences frame our exploration of co-creation and implementation. While participants in each country co-created the interventions with their own concerns in mind, the broad topics they identified were the same in both countries (see SM5 for further details). This highlights that, despite considerable contextual variation, certain mental health concerns among adolescents appear to be universal.

Although vastly different environments, both countries’ health systems cannot meet the youth mental health needs. In a nationally representative survey of 700 Tanzanian secondary school students, 41% demonstrated an elevated level of mental ill-health in the previous 6 months (Nkuba et al., 2018). WHO (2020) data indicate that Tanzania had ~38 psychiatrists or 0.07 per 1,00,000 people in Tanzania, with psychiatric nurses providing the bulk of services; these nurses are operating at full capacity, with a ratio of 0.85 per 1,00,000, and have limited training in youth mental health. Mental health infrastructure is also lacking, with few mental health hospitals, psychiatric units and community-based mental health care programs, especially in rural and more remote areas (Mwambingu et al., 2019; WHO, 2020; León-Himmelstine et al., 2021).

In Vietnam, a 2022 study found that 21.7% of adolescents had faced mental health problems in the previous 12 months, with anxiety being the most prevalent (18.6%), followed by depression (4.3%) (Institute of Sociology et al., 2022). The same study found that only 8.4% of adolescents had accessed mental health services. Similar to Tanzania, there are few psychiatrists – 900 according to one estimate (Cuong, 2017) covering a population of ~100 million. Most psychiatrists focus on treating severe mental disorders, have limited expertise and training in youth mental health and are concentrated in large cities (UNICEF, 2018). In Vietnam, student mental health services are offered through school-based psychological counseling units and several telephone hotlines. However, counseling units are not available in all schools, their quality has been questioned and uptake – like that of the hotlines – remains low due to stigma and concerns about confidentiality (UNICEF, 2018).

This article describes the results of a 3-year project guided by three research questions (RQs):

What are the key drivers and protective factors of adolescent (10–19 years) mental health in two regions in Tanzania (Morogoro and Mwanza) and two cities in Vietnam (Vinh City and Nha Trang City)?
What mental health services are available to adolescents, and how accessible are they?
(How) can we co-create an intervention to address adolescent mental health in Tanzania and Vietnam?

Implicit in our study is a socioecological framing, which draws on Bronfenbrenner’s (Bronfenbrenner, 1977) ‘nested circles’ framework to explore how drivers of youth mental (ill)health operate at multiple levels. Proximal influences include family, peers and friends, while more distal circles encompass the school environment and wider community. The outer-level circles also capture the service and policy environment, together with the social and gender norms that underpin behaviors and attitudes, and constitute a central concern of our study. These interrelated levels are explored through the various outputs of our 3-year project.^1^

## Methods

This mixed-methods, quasi-experimental study, which included intervention and comparator groups, followed an explanatory sequential design (Creswell and Clark, 2017): qualitative research built on the quantitative surveys to explore further and triangulate emerging findings. The qualitative component also elicited greater nuance on sensitive issues, such as stigma and access to services (RQ2). In addition, survey data (i.e., the Strengths and Difficulties Questionnaire; see below) informed the selection of some respondents for the qualitative study.

### Quantitative surveys

The research teams conducted pre- and post-intervention surveys in 2021 and 2023, respectively. These were self-administered by adolescents (aged 10–19 years) across four participating schools in Tanzania and eight in Vietnam (see SM2 and Supplementary Table S2).

#### Sampling of schools and classrooms

The study employed a two-stage stratified cluster sampling design. In the first stage, schools were purposively selected to ensure diversity in level and location. In each region in Tanzania, the in-country project coordinator selected two schools in an urban/peri-urban and a rural area, respectively, from a list of schools possessing a minimum of 10 working computers and teachers with basic information and communications technology skills. In each city in Vietnam, the district authority selected two urban and two suburban schools. In a second stage, classrooms were selected randomly within each school (20 in Vietnam and 10 in Tanzania). The number of classrooms was determined to achieve a sample size with sufficient statistical power, based on 95% confidence intervals (95% CIs) and a 5% standard error.

The intervention took place at every school, with the comparator group consisting of nonparticipating youth in the same schools. The intervention (clubs) was open to all students, introducing the possibility for selection bias; however, random allocation was not feasible given the study design and implementation context. We sought to mitigate this bias by computing sample weights to readjust the demographic composition of the intervention group to that of the full baseline (see SM2). The intervention (clubs) may have generated spillover effects that benefited the wider school, thereby introducing potential contamination of the comparator groups. However, such spillover is likely to make any observed positive intervention effects even more noteworthy. Panel data are available in Vietnam for both intervention and comparator groups, and in Tanzania for the intervention group; for the Tanzanian comparator group, only cross-sectional data are available.

#### Adjustments to sampling protocol

Because intervention participation was not limited to students in the original baselines, a second baseline survey was conducted in both countries. In Tanzania, it covered students who had not taken the original survey but joined the intervention, while in Vietnam – due to COVID-19-related school closures – it was administered to all intervention participants (see SM2 for details). With ~24 months separating the baseline and endline surveys, just over half of the students who participated at baseline (54% in Tanzania and 52% in Vietnam) were no longer present at endline. Accordingly, our endline included many ‘replacement’ students. In Tanzania, we treat the comparator samples at baseline and endline as cross-sections, and our intervention participants as a panel. In contrast, the larger sample size in Vietnam enabled the inclusion of a panel subsample that comprised participants from both the intervention and comparator groups.

#### Core mental health measures

The survey included six core scales: (i) the Emotional Literacy Scale (ELS) (Carnegie School of Education, 2018), which assesses knowledge and attitudes regarding mental health; (ii) the Mental Health Promoting Knowledge scale (MHPK-10) (Bjørnsen et al., 2017); (iii) Attitudes Toward Seeking Professional Psychological Help (ATSPPH) (Elhai et al., 2008); (iv) the Kidcope scale (Spirito et al., 1988), which assesses informal coping strategies; (v) the Strengths and Difficulties Questionnaire (SDQ) (Goodman et al., 2000), which assesses emotional and behavioral difficulties; and (vi) the WHO-5 index (Topp et al., 2015), which assesses subjective psychological well-being (see SM1 for details).

#### Analytical strategy

Analyses were conducted separately for Tanzania and Vietnam to reflect differences in study design.

For Tanzania, analyses were conducted in Stata. Within-group pre–post results are presented with unadjusted *p*-values for descriptive purposes. Because the comparator group was a repeated cross-section with mostly different participants at baseline and endline (see SM2), a difference-in-differences (DiD) regression analysis was applied to assess the intervention’s effects. For each of the eight core mental health outcomes, weighted regressions estimated within-group pre–post changes and the adjusted DiD between intervention and control groups, controlling for key sociodemographic variables (age, gender, region, socioeconomic status (SES) and the experience of hunger in the previous year). The intervention effect – captured by the interaction term between time (baseline/endline) and group (intervention/comparator) – isolates the specific impact of being in the intervention group at endline. Robust standard errors were used to account for heteroskedasticity. Standardized effect sizes were calculated by dividing the adjusted intervention effect by the residual standard deviation from the full regression model, yielding adjusted Cohen’s *d* as an indicator of practical significance.

For Vietnam, analyses were conducted using Statistical Package for Social Science (SPSS). Significant differences across the core mental health measures and sociodemographic characteristics are reported based on independent-samples *t*-tests, with adjusted *p*-values, based on provided to account for multiple comparisons. Given the panel design, one-way analysis of covariance (ANCOVA) was used to measure the intervention’s effects, controlling for baseline scores of each mental health outcome in the comparator and intervention groups. Where sample sizes allowed, additional covariates (gender, age and socioeconomic status) were included to provide a deeper understanding of factors associated with intervention effects. Tests for homogeneity of variance (Levene’s test) and normality confirmed that all ANCOVA assumptions were met. Partial eta-squared (*η*^2^) is presented as a measure of effect size – representing the proportion of variance in the dependent variable explained by each predictor, controlling for other covariates.

Adjusted p-values for the DiD and ANCOVA models—calculated using the Benjamini–Hochberg false discovery rate procedure—are reported in footnotes 3 and 4. These values are provided for transparency; however, we do not place analytic emphasis on them given the exploratory nature of the study, our ongoing examination of the interrelationships among the mental health indicators, and our awareness of Rubin’s (2024) caution against undue “statisticism.”

### Qualitative study

The surveys were complemented by in-depth interviews (IDI), key informant interviews (KIIs), focus group discussions (FGDs) and family case studies (FCSs) with adolescents, parents, teachers and service providers. IDIs with adolescents elicited personal experiences and perceptions; FGDs provided insights into community perspectives; FCSs provided information about family dynamics and other family members’ perceptions of the mental health of the adolescent in question; and KIIs with teachers and service providers helped explain the service environment. Given our implicit socioecological framing, such interviews also reflect the importance of triangulation and understanding how adolescent mental health is driven by a combination of individual factors, relationships with family and peers, as well as the school and the wider service environment.

#### Recruitment for adolescent IDIs

Two approaches were used to recruit adolescent participants at baseline for the IDIs. First, schoolteachers in each selected school provided lists of adolescents disaggregated by age, distinguishing mid-adolescence (10–15 years) and older adolescence (16–19 years) and gender; they also identified students who were already in leadership roles and could potentially take leadership roles in the intervention (see SM5). The research teams randomly selected IDI participants from each list. Second, respondents were selected from the survey based on their SDQ scores: those exhibiting relatively high levels of internalizing issues (e.g., depression or anxiety) were invited to take part in IDIs. At endline, adolescents who had taken part in the intervention were selected for IDIs, some of whom had taken a leadership role and some of whom had relatively high SDQ scores.

#### Recruitment for FGDs, FCSs and KIIs

FGDs, consisting of 6–18 participants, were carried out separately with adolescents, mothers and fathers. The adolescent FGDs combined girls and boys, as suggested by the country research teams. Adolescent participants were recruited with the support of teachers, and parents were recruited through snowballing, facilitated by adolescent participants. At endline, adolescent FGDs comprised a mix of intervention participants and student leaders who had not taken part.

Participants in the FCSs, where different generations of a family are interviewed, were also recruited through the interviewed adolescents; they included mothers, fathers, siblings and/or grandparents. Some FCSs included just one other family member, while others included several.

Finally, KIIs with those working on or interested in youth mental health were recruited via the schools (for teachers) and via local authorities, government line departments, non-governmental organizations and community or youth groups.

In Tanzania, a total of 93 interactions (including IDIs, FGDs, FCSs and KIIs) were conducted at baseline and 98 at endline; in Vietnam, a total of 92 interactions were conducted at baseline and 78 at endline. The concept of thematic saturation was applied in both countries during both rounds of data collection. A further breakdown of the number and characteristics of respondents at each site in both countries is provided in the Annexes of Samman et al. (2023) and Samuels et al. (2023).

#### Analytical strategy

The qualitative data were analyzed using thematic analysis and an inductive approach, informed by the principles of interpretive phenomenological analysis (Brocki and Wearden, 2006), whereby both the study participants’ and research teams’ lived experiences and positionality are taken into account during data analysis and interpretation. After transcribing and translating verbatim the recorded interviews and FGDs, the transcripts were uploaded to MaxQda, which was used for data management, coding and theme generation (Braun and Clarke, 2006). A six-phase thematic analysis framework was used to analyze the data. The analysis was led by two experienced qualitative researchers who first read the transcripts to gain familiarity with the data, then jointly generated a preliminary coding framework. This framework was subsequently discussed and refined through meetings with the qualitative research teams in each country. Using the finalized codebook, the two qualitative researchers independently coded the data, with both researchers double-coding 10% of the transcripts to enable discussion and resolution of any discrepancies and to ensure inter-coder reliability. Additional codes and subcodes were added inductively throughout this process. Next, the researchers grouped similar codes and generated themes, which were reviewed and refined with input from the qualitative research teams in each country.

Data were collected by research teams from the Tanzanian Training Centre for International Health and the Vietnam National University of Education, supported remotely by an ODI-led international team.

#### Ethical considerations

Ethical clearance was obtained from the ODI Research Ethics Committee (ref P000005), where the Principal Investigator was based at the time. In Tanzania, ethical approval was obtained via the National Institute for Medical Research. In Vietnam, permission was granted via the Provincial Department of Education and Training of Vinh City and Nha Trang City (see SM4 for further details).

## The co-creation process and co-created initiatives

The design of the intervention reflected a co-created approach (Vargas et al., 2022), involving collaboration from the outset with key stakeholders, including adolescent end-users. This process encompassed problem identification (via baseline studies), solution design, support to implementation, ongoing feedback and adaptation and input into acceptability.

Co-creation workshops, facilitated by the country research teams and lasting ~3 days, were held in each school, involving adolescents, teachers, parents and local authority representatives. Workshops combined research team presentations, small-group work and plenary discussion. This process produced an intervention prototype, which the research team finalized following the workshops, then shared with adolescents to ensure the final version remained faithful to their design (Myers and Samuels, 2022).

In both countries, the initiative comprised hybrid digital and in-person elements (Samman et al., 2023; Samuels et al., 2023). In Tanzania, the in-person component combined discussions and debates with sports, games, poetry writing and poster making. The digital component comprised a computer-based learning platform that included a digital library with interactive mental health resources and a mood-tracking application. In Vietnam, the in-person portion also combined discussion and games, while the digital component included a mood-tracking application and a Facebook page. In both countries, local research teams worked closely with the facilitators (teachers in Tanzania and local psychologists in Vietnam) to prepare the sessions and manuals in Swahili and Vietnamese, respectively. Further details of the co-creation process and the co-created interventions can be found in SM5.

## Results

This section presents findings from the mixed-methods study, highlighting several domains where the co-created initiative had positive effects. Tables 6.1 and 6.2 in SM6 summarize key characteristics of the survey respondents in each country at baseline and endline, while Table 7.1 in SM7 summarizes qualitative baseline and endline themes and subthemes. Given space constraints, we present key findings from the quantitative survey and describe how the qualitative research adds additional nuance and context. While the qualitative study sought to explore differences by gender and age, no significant patterns emerged beyond those highlighted in the text.

## Effects on mental health literacy: Knowledge and attitudes

In both countries, the initiative was associated with improved mental health literacy, measured by two quantitative scales, the ELS and MHPK-10. In Tanzania, participants registered gains of 15.9% for the mean ELS score (*p* = 0.000) and 9.7% for the mean MHPK-10 score (*p* = 0.000), while gains for the comparator group were ~6% for each scale (*p* = 0.000). Regression analysis indicated that the increase in the mean ELS score for the intervention group was 9.5% greater than that of the comparator group, after adjusting for key sociodemographic differences between the two groups (*b* = 0.24, 95% CI: 0.067–0.417, *p* = 0.007; adjusted Cohen’s *d* = 0.57). However, for the MHPK-10, the additional increase of 0.53% for the intervention group relative to the comparator group was not statistically significant (*b* = 0.016, 95% CI: −0.170 to 0.202, *p* = 0.866; adjusted Cohen’s *d* = 0.03).

In Vietnam, in both Nha Trang and Vinh, the mean ELS score showed statistically significant increases of 8% (*p* = 0.021, FDR adjusted p = 0.030) and 5% (*p* = 0.030, FDR adjusted p = 0.030), respectively, for the intervention group, and no significant changes in the comparator group. Among participants, the effect was particularly significant among boys (8%; *p* = 0.008, FDR adjusted p = 0.008), younger students aged 12–15 years (11%; *p* = 0.003, FDR adjusted p = 0.006) and those with low SES (17%; *p* = 0.001, FDR adjusted p = 0.003). One-way ANCOVA using the panel data found a small but significant intervention effect in ELS levels when controlling also by gender (*F*(1, 330) = 6.411, *p* = 0.012, *η*^2^ = .019). The intervention group improved its mean ELS score by 5.3% compared to the comparator group (95% CI: 1.2–9.4%). The mean MHPK-10 score also registered an increase, but this was statistically significant and positive only among male students (9%, *p* = .004, FDR adjusted p = 0.008) and those with low SES (12%, *p* = 0.014, FDR adjusted p = 0.042). The ANCOVA finds a small but not significant increase in the mean MHPK-10 score of 2.4% (95% CI: −1.2 to 6%) for the intervention group (*F*(1,277) = 1.734, *p* = 0.189, η^2^ = .006).^2^

The qualitative studies in both countries found similar benefits, including an improved understanding of drivers of mental ill-health.

*I did not know quarrels and insults could cause mental illnesses, but the program enhanced my understanding and I decided to share this with my family at home.* (12-year-old girl, Mwanza)

Participating adolescents reported that the project taught them how to assess and deal with their own mental health, to see the importance of social connection and to have a more positive mindset.

*‘There was a lesson about anxiety disorder. I was a person with severe anxiety disorder… However, when I learnt more about this disorder, I gradually realized and changed to a more positive mindset’.* (14-year-old girl, Vinh)

The initiative also positively affected parent–child interactions and had spillover effects on teachers, parents and other stakeholders who reported improvements in their own understanding of mental health.

*… I have come to know about mental health after attending that seminar. I now understand that it is a big problem in our society, but many people do not know about it.* (Administrator and chaplain, Mwanza)

Turning to attitudes toward mental health services and help-seeking behavior, measured by the ATSPPH scale, for Tanzania, the survey data show an increase in positive attitudes among intervention participants of 7.9% (*p* = 0.006) compared with 1.5% (*p* = 0.296) for the comparator group. After adjusting for key sociodemographic differences, the intervention group showed an estimated 9.1% increase (*b* = 0.272, 95% CI: 0.041–0.503, *p* = 0.021; adjusted Cohen’s *d* = 0.50) relative to the comparator group, indicating a moderate effect. In Vietnam, no statistically significant changes were observed in the ATSPPH scale, except for an increase of 17% in the mean score among students with a low SES (*p* = .001, FDR adjusted p = 0.030). The ANCOVA test showed no statistical significance (*F*(1,276) = 3.857, *p* = 0.051, *η*^2^ = .014).

In both countries, the qualitative study showed improvement in the attitudes of participating adolescents and parents regarding mental health services, due to an increased understanding of the potential benefits. Nonetheless, adolescents and parents/adults reported continuing stigma around service access, particularly in Vietnam.

‘*I’m not comfortable going to that hospital [psychiatric hospital]. I worry that if I take my child there, their friends will make fun of them, saying they are crazy. I wish there will be a private service. People will think we are crazy if they see us go to a psychiatric hospital. People have a problem with psychiatric hospitals …*’. (Group discussion with parents, Nha Trang)

In Vietnam, a gendered narrative was evident, persisting also after the intervention. Boys and girls associated mental health issues with stereotyped ideas around ‘feminine’ attributes and observed that girls were most susceptible to mental ill-health because they are ‘born as the weaker gender’ and ‘more sensitive than boys’. In Tanzania, the project team observed that more males than females sought mental health services; they suggested that girls were not taken as seriously when expressing their emotions and were sometimes labeled as ‘hysterical’.

*‘They still think people with depression or those having negative thoughts are weak and are cry-babies.’* (14-year-old girl, Vinh)

## Effects on mental health: Drivers and protective factors

Two measures were used to quantify mental health: the SDQ and the WHO-5 Well-Being Index. Turning first to the SDQ, in Tanzania, two dimensions emerged: (i) mental health difficulties – emotional symptoms, conduct problems, hyperactivity and peer problems; and (ii) prosocial behavior, or behaviors that show empathy and the ability to relate well with others. When comparing baseline and endline, adolescents in the intervention group registered a 9.2% rise in the mean score on the prosocial behaviors subscale (*p* = 0.006) and a similar (but not statistically significant) decline of 8.75% in the mean score on the mental health difficulties subscale (*p* = 0.290). Among the comparator group, the change in the prosocial behaviors score was negligible and not statistically significant, while the mean score on the mental health difficulties subscale increased by 9.3% (*p* = 0.043). Regression analysis suggested an 8.6% increase in the mean prosocial behavior score for the intervention group relative to the comparator group over time (*b* = 0.066, 95% CI: 0.002–0.130, *p* = 0.043; adjusted Cohen’s *d* = 0.38), indicating a modest.

In Vietnam, the SDQ identified three dimensions: (i) emotional problems, (ii) behavioral problems and (iii) prosocial problems. The evaluation revealed little or no change in the intervention group across the three dimensions. We hypothesized that the initiative may have protected participant mental health, given small increases in the mean score on the emotional problems subscale for the comparator group (3%, *p* = 0.006), but this was not sustained by the ANCOVA test using the panel data (95% CI: −3.5 to 7.9, *F*(1, 325) = 0.585, *p* = 0.445, *η*^2^ = 0.00).

Turning to the WHO-5 Well-Being Index, in Tanzania, between baseline and endline, the mean score for the intervention group increased 10.2% (*p* = 0.006), compared with 5.5% for the comparator group (*p* = 0.009), though the increase of 7.15% among the intervention group relative to the comparator group was not statistically significant once controlling for sociodemographic differences (*b* = 0.308, 95% CI: −0.071 to 0.686, *p* = 0.111; adjusted Cohen’s *d* = 0.28). In Vietnam, the only significant increases in well-being were observed among intervention group boys (9% increase in the mean score, *p* = 0.043, FDR adjusted p = 0.086) and those with low SES (23%, *p* = 0.005. However, the ANCOVA test did not find any statistically significant effect (95% CI: −8.2 to 9.1, *F*(1, 276) = 0.916, *p* = 0.916, *η*^2^ = 0.00).

At baseline, a range of drivers of mental ill-health were identified qualitatively (León-Himmelstine et al., 2021; Samuels et al., 2022). Drawing on a socioecological framing, at an individual level, these included poor self/body image (especially for girls in Vietnam), a lack of friendships and loneliness/isolation, which increased during COVID-19, especially in Vietnam (Samuels et al., 2021). At a household level, they included poverty (especially in Tanzania), lack of family support or poor intra-household dynamics and gender norms – especially in Tanzania, where, for instance, child marriage for girls is often expected and increased during COVID-19 (Leon-Himmelstine et al., 2021). Beyond the household, school-level drivers included peer and academic pressure, violence/bullying from teachers and excessive use of technology, especially in Vietnam. Protective factors were mirror images of drivers and included positive self-image and having friends and supportive family relationships.

*‘‥I think being together with my family and … being able to see my friends every day, that’s what makes me happy’.* (Group discussion with 13-year-old girls, Nha Trang)

In both countries, while the drivers of mental ill-health remained unchanged, participating adolescents reported a greater understanding of these factors and improved capacity to address them, reframe negative self-perceptions and focus on sources of happiness. This translated into stronger relationships with parents and friends, improved school performance and a greater ability to cope with academic pressure.

*I started spending more time with my friends after I joined the mental health program. Before I joined the mental health club, I was going home immediately when the bell rang, but now I do not leave immediately, I share stories with my friends.* (17-year-old boy, Morogoro)

## Effects on coping strategies

Adolescents’ coping strategies were measured with the Kidcope scale, wherein three coping subscales were developed: active (e.g., problem-solving and cognitive restructuring), avoidant (e.g., social withdrawal) and emotional/expressive (e.g., anger and seeking support online). In Tanzania, the largest changes were in the active coping subscale: the mean score active coping among adolescents in the intervention group increased 21.7% (*p* = 0.000), compared to a 2.5% increase in the comparator group (*p* = 0.264); the regression analysis indicated a statistically significant rise of 15.6% in active coping for the intervention group, relative to the comparator group (*b* = 11.85, 95% CI: 3.45–20.25, *p* = 0.006; adjusted Cohen’s *d* = 0.55).

In Vietnam, the initiative appears to have prevented an increase in emotion-focused coping. Although for the intervention group the reduction in the mean score on the emotion-focused coping subscale (5.7%) was not statistically significant (*p* = 0.087), the average mean score of the comparator group rose by 23% (*p* = 0.001). The ANCOVA test confirms a small but statistically significant effect of 14.4% (95% CI: −27.5 to −1.3, *F*(1, 309) = 4.664, *p* = 0.032, *η*^2^ = 0.015). The ANCOVA also found a small but significant effect of 8.6% decline in active coping (95% CI: −16.2 to −1.0, *F*(1, 324) = 5.001, *p* = 0.026, *η*^2^ = .015), and a marginally significant decline in avoidant coping (95% CI: −41.7 to 0, *F*(1, 322) = 3.864, *p* = 0.05, *η*^2^ = .012). Stronger and statistically significant effects were observed at an item level; for example, selected avoidant coping strategies, such as a tendency to self-blame, declined over 20 percentage points (*p* < 0.05).

The qualitative studies in both countries identified various positive (active) and negative (avoidant/emotional) coping strategies adolescents adopted to deal with challenging situations. Positive strategies included talking to/confiding in someone and using distractions – sports, listening to music, reading, interacting with friends and praying (especially in Tanzania). Negative or avoidant strategies included sleeping, isolation and skipping meals. Adolescents also engaged in risky behaviors including self-harm, emotional eating, suicidal thoughts, violence, bullying and substance abuse. Although similar positive and negative coping strategies were recorded at endline, adolescents and adults in both countries reported a reduction in negative strategies and a turn to more positive ones.

*Initially, some of the children liked to self-isolate but after attending the sessions and becoming aware, whenever they find their peer in that situation, they help him or her.* (Teacher, Morogoro)

In both countries, participating adolescents who had previously self-harmed reported having stopped. In Vietnam, they instead confided in others and sought help when needed. Others highlighted improved emotional regulation, stress management, empathy and patience.

*‘…I had… hurt myself. But… now not anymore. Now, I will find a friend to talk to cope with my sadness.’* (17-year-old girl, Vinh)

## Discussion

The initiative had positive effects in both countries. The quantitative analysis showed higher benefits among the intervention group in Tanzania for all our key mental health scales, with regression analysis, indicating that (for all measures, except the MHPK-10 and WHO-5) benefits persisted once controlling for key differences between the comparator and intervention groups.^3^ Based on the ELS, the ATSPPH scale and the KidCope Active Coping subscale, the effect sizes fall within the medium range; and for the two SDQ subscales and the WHO-5 scale, they are classified as small to medium.

For Vietnam, the results are more mixed: ANCOVA testing using the panel data uncovered positive effects of the intervention on emotional literacy, as well as declines in emotion-focused and avoidant coping and an increase in knowledge of where to seek mental health information.^4^ However, active coping – the more positive form of coping – did not increase significantly. Moreover, the analysis did not identify intervention effects relating to knowledge of promoting mental health, attitudes toward professional help-seeking, nor on our two mental health status measures (the SDQ and WHO-5).

The qualitative analysis also reported improved emotional literacy and mental health status. However, this was the case for both countries, with participants observing they had adopted more beneficial coping strategies to deal with mental ill-health. Adolescents in both countries also linked their participation to increased empowerment and confidence, largely because they were involved in co-creating the intervention and subsequently being part of it. This also translated into better social relationships among peers and between adolescents and adults, including parents and teachers.

While the study did not aim to compare findings across the two countries, it is nevertheless interesting to explore briefly why there may be differences in the intervention effects and why the quantitative findings in Vietnam were less clear than in Tanzania. One explanation is that, although session content was similar, delivery modalities differed (see SM5). The Tanzanian team’s greater experience with co-creation and participatory research may also have enhanced the intervention’s acceptability and, in turn, its outcomes.

The cultural context might also help explain why active coping did not increase among the Vietnamese students. It is possible that the hierarchical structures prevalent within Vietnamese society limit students’ opportunities to solve their own problems, leading them to rely on adult direction. Relatedly, communication between students and adults, including parents, is limited, and adolescents – as also found in the qualitative study – do not actively seek out people to support them. Consequently, shifts toward more active coping strategies may take more time to emerge.

Notwithstanding these varying effects, this represents the first SBMH initiative in both countries in which the content and mode of delivery were co-created largely with adolescents. The intervention focused on addressing well-being rather than treating mental disorders, adopting a universal/whole school approach within which some targeting occurred with support from teachers and through student self-selection. The adaptive nature of the intervention was distinctive, with regular check-ins enabling adjustments to aspects that were not working. The AMP study also extended its focus to areas beyond urban centers, which remain relatively neglected in terms of mental health-related activities and service provision in both countries.

Interestingly, while the intervention was co-created in each country to reflect local needs and priorities, the topics deemed important to cover were the same. Despite contextual differences, this suggests some universality in the mental health-related challenges youth face in transitioning from childhood to adolescence.

Unplanned spillover effects were also evident: nonparticipating adolescents and teachers started discussing mental health, including in school assemblies, and a clinic adjacent to one school in Tanzania started providing mental health services. While spillover effects cannot be attributed to the project alone, these findings, along with those from other studies, suggest the need to purposefully consider any broader multiplier effects (Jabeen, 2016; Francetic et al., 2022).

The lack of positive change for all groups and measures may indicate that the learnings need more time to embed. Going forward, policy and research recommendations include: (i) working with ministries of Education to embed and appropriately resource such an approach in school curricula; (ii) engaging institutions (government, nongovernment, youth groups and traditional and religious organizations) to increase mental health literacy; (iii) building capacity of lay mental health workers to support delivery of youth targeted mental health services; (iv) advocating for universal SBMH initiatives (with some targeting and self-selection) that are co-created with end users; and (v) more research including feasibility and effectiveness trials to strengthen the evidence base for implementing, embedding and scaling-up such approaches in LMICs.

## Limitations

We identify several important limitations. Our purposive approach to sampling schools sought to maximize diversity, but in the absence of a complete sampling frame, we cannot conclude that findings are regionally representative. Other potential limitations include selection bias among intervention participants, contamination of the comparator group and clustering effects inherent in school-based interventions.

Our efforts to control for self-selection bias by constructing sampling weights to readjust the demographic composition of the intervention group to that of the full baseline were only partially successful (see SM3). However, the nature of the intervention (self-selection into clubs), along with ethical and practical challenges in recruiting control schools, and financial constraints meant that alternative designs (e.g., randomized or cluster randomized control trials) were not feasible.^5^

Selection bias was also present in the qualitative sample. Most adolescent respondents were selected randomly by a list provided by teachers, including some who were in leadership positions, while the research team purposefully selected others with relatively low SDQ scores. As a result, the sample is not representative of the broader student population. Nevertheless, its diversity renders it appropriate for understanding the range of experiences related to adolescent mental health.

The final limitation relates to COVID-19. The pandemic did not affect the baseline survey because no lockdown measures were in place in either country at that time. Qualitative data collection activities were similarly unaffected; however, the content of the interviews at baseline was reduced slightly to account for participants’ time constraints. The number of FGD participants was limited to five to eight, and they were kept apart to control disease transmission. We cannot quantify how COVID-19 affected participant responses, although some studies conducted as part of this project suggest significant mental health effects (Chakraborty et al., 2021; Leon-Himmelstine et al., 2021; Samuels et al., 2021). In Tanzania, the team was unable to probe deeply into the mental health effects of COVID-19 as this was a sensitive topic under the former government, leading students and adults to respond cautiously.

## Conclusion

Awareness of the need to address the youth mental health burden in LMICs is growing, including in Tanzania and Vietnam. Universal SBMH initiatives offer a potential response, including those co-created with adolescents themselves and partly run by them. This does not detract from the need to invest in mental health training – including that of lay mental health providers – which is an effective strategy to overcome human resource pressures, including in LMICs and in rural areas (Rebello et al., 2014; Hoeft et al., 2018). This strategy is being integrated into a subsequent phase of this (M-BRIGHT),^6^ which extends, through co-adaptation, the intervention to other sites in Vietnam and to Cambodia. A central component of M-BRIGHT is, therefore, to explore ways to embed the approach and thereby ultimately strengthen the adolescent mental health delivery system.

## Supporting information

10.1017/gmh.2025.10100.sm001 Samuels et al. supplementary material Samuels et al. supplementary material

## Data Availability

Data available on request due to privacy/ethical restrictions.

## Footnotes

1 For further details of the various outputs, see: https://odi.org/en/search/?project=Addressing+the+mental+health+needs+of+adolescents+in+Tanzania+and+Viet+Nam+through+the+co-creation+of+digital+and+non-digital+solutions&sort=date.

2 The lack of statistical significance persisted after controlling for socioeconomic status and gender. The small size of the panel data sample may have affected the likelihood of finding a significant result.

3 FDR correction for multiple comparison provides a more conservative result ATSPPH (p = 0.055) and prosocial behaviours (p = 0.068).

4 FDR correction for multiple comparison provides a more conservative result with no significant p value (p = 0.120) for either emotional literacy, emotion-focused and avoidant coping.

5 As of November 2024, a follow-up study is underway in Cambodia and Vietnam to assess the feasibility of a full cluster randomized control trial.

6
https://www.qmul.ac.uk/wiph/centres/centre-for-public-health-and-policy/global-public-health-unit/m-bright/.

## Supplementary material

The supplementary material for this article can be found at http://doi.org/10.1017/gmh.2025.10100.
