# Supplementary material for: Promoting adolescent mental health in Tanzania and Vietnam through a co-created universal school-based initiative: Findings from a mixed method study
Source: Glob Ment Health (Camb). 2025 Dec 1;12:e142. doi: 10.1017/gmh.2025.10100 (PMC12720375; doi:10.1017/gmh.2025.10100)
Supplement: Samuels et al. supplementary material [file S2054425125101003sup001.docx]

**Supplementary Materials**

Contents

[1. Core mental health measures 1](#_Toc212459690)

[2. Quantitative sample size and composition by school at each baseline and at endline 5](#_Toc212459691)

[3. Selection bias and response bias in the survey 7](#_Toc212459692)

[4. Details of ethics process 8](#_Toc212459693)

[5. Existing literature on SBMH in Vietnam and Tanzania and details of the co-created process and intervention 9](#_Toc212459694)

[6. Number of survey respondents and their characteristics at baseline and endline, Tanzania and Vietnam 17](#_Toc212459695)

[7. Qualitative themes and sub-themes 19](#_Toc212459696)

[8. Study phases / timeline 20](#_Toc212459697)

## Core mental health measures

This section discusses the scales we selected to measure key constructs in the survey: i) the Emotional Literacy Scale (Carnegie School of Education 2018); ii) the Mental Health Promoting Knowledge scale (MHPK-10) (Bjørnsen et al. 2017); iii) Attitudes Toward Seeking Professional Psychological Help (Elhai et al. 2008); iv) the Kidcope scale (Spirito et al. 1988); v) the Strengths and Difficulties Questionnaire (SDQ) (Goodman et al. 2000); and vi) the WHO-5 index (Topp et al. 2015). It situates them in the literature, describes the piloting and psychometric testing undertaken to establish their validity and reliability, and context-specific adaptations made to the Kidcope scale.

The survey included two measures of mental health awareness: The first is the Emotional Literacy scale, developed by Carnegie School of Education (2018), which was used to inform a school-based mental health intervention in Cambridge, UK. The scale is, in turn, an adaptation of the Mental Health Literacy Scale (O'Connor and Casey 2015) which aims to assess both stigma and knowledge concerning mental health. The adaptations removed questions asking about specific, and often complex, mental health disorders as well as questions that were inappropriate for the age group e.g. around employment and added questions asking about the participants’ sense of their own resilience, strategies for stress and social media use Carnegie School of Education 2018:8)

The second mental health awareness measure is the Mental Health Promoting Knowledge scale (MHPK-10)(Bjørnsen et al. 2017). This measures knowledge of what is important for good mental health and was developed and validated among Norwegian upper secondary school students. This latter scale fills an important gap as it is the first to quantify ‘knowledge of good or positive mental health’ as opposed to mental health disorders, stigma or health-seeking behaviour (Wei et al. 2015, cited in Bjørnsen et al. 2017: 2).

We assess help-seeking behaviour by exploring student attitudes towards seeking professional help to address mental health concerns as well as informal coping mechanisms. We measure the former using the Attitudes Toward Seeking Professional Psychological Help scale-Short Form (ATSPPHSF)(Fischer and Farina 1995); building on the original ATSPPH scale devised by Fischer and Turner, 1970), a widely cited measure of mental health treatment attitudes. Per Elhai et al. (2008: 321), this is the only ‘standardized instrument assessing mental health treatment attitudes’ that ‘has been both psychometrically examined and used in a sizeable number of studies’.

We measure a diverse range of informal coping strategies using the Kidcope scale (Spirito et al. 1988) with minor contextual adaptations based on scale piloting and research team inputs. The scale was originally designed to measure children’s use of 10 behavioural and cognitive coping strategies following hospitalisation but has subsequently been used widely to assess coping with respect to a range of stressors (Powell et al. 2019). The strategies included are distraction, social withdrawal, cognitive restructuring, self-criticism, blaming others, problem-solving, emotional regulation, wishful thinking, social support and resignation to cope with a major stressor.

In the survey, mental health is measured through two key scales. The Strengths and Difficulties Questionnaire (SDQ) - see https://www.sdqinfo.org/a0.html - evaluates emotional and behavioural difficulties among youth. The WHO-5 - See https://www.who.int/publications/m/item/WHO-UCN-MSD-MHE-2024.01 - is ‘among the most widely used questionnaires assessing subjective psychological well-being’ (Topp et al., 2015: 167) in children aged nine and over; it also has adequate validity in screening for depression. These two measures therefore provide complementary insights into mental (ill)health. Both have been widely validated in diverse settings and among varied populations globally, including in Tanzania (for the SDQ, see (Dow et al. 2016), (Hermenau et al. 2011; Hermenau et al. 2015); (Nyangara F 2009); (Hoosen et al. 2018); for the WHO-5, see (Nolan et al. 2018).

In both countries, the questionnaire and psychometric scales were tested and refined after piloting of the survey (for details of testing that took place prior to and following baseline data collection, see (León-Himmelstine et al. 2021), Annex 3). In Tanzania, the survey team conducted two rounds of pilot data collection with secondary school students in Morogoro. The first round included 100 students, the second had 80 students. In Vietnam, the survey team conducted a pilot survey with 185 secondary students from a secondary school in Hanoi and an upper secondary school in Hai Phong following a convenience sampling.

The team input data from the questionnaires using tables programmed with the Open Data Kit application. On review of the scales for their psychometric properties including reliability (internal consistency),^[[1]](#footnote-1)^ criterion validity and construct validity, the team made some improvements to the questionnaire. The team re-tested the psychometric properties of each scale following baseline data collection and once again at endline (for Tanzania, see Samman et al. 2023, Annex Tables Data, Tables 5.2–5.5 tabs; for Vietnam, see Samuels et al. 2023, Annex A2b and A2c).

We also conducted exploratory factor analysis at baseline and endline, and for the pooled dataset. In comparing baseline and endline, we used a pooled dataset including both baseline and endline data to establish thresholds that we then applied to both rounds. For some scales, to maximise construct validity and reliability, we only retained data for scale items that were loading as expected in the exploratory factor analysis and excluded those that would increase Cronbach’s alpha if the item was deleted. This enabled us to construct measures that were most attuned to the context where the survey was administered, albeit at the expense of comparability with other studies conducted in Tanzania, Vietnam or elsewhere. For Tanzania, Samman et al. 2023 (Annex Tables Data, Tables 5.6 and 5.7) provide full results of the exploratory factor analysis conducted for the SDQ and Kidcope scales respectively, using this pooled dataset. For Vietnam, Samuels et al. 2023 provide this information for Vietnam. In the case of Kidcope, we also present the eventual 15 item solution, reduced from the 22 items in our questionnaire (Table A.1).

Tables 5.8 and 5.9 in Samman et al, 2023, Annex tables data and Samuels et al. 2023, Annex 2a) provides full details of the quantitative sample at baseline and at endline, respectively.

**Table S.1. Contextual adaptations made to the 15-item Kidcope scale**

| **Note** | **Coping group** | **Item #** | **Statement** |
| --- | --- | --- | --- |
| * | Distraction | 1 | I just tried to forget it |
| * | Distraction | 2 | I did something like watch TV, listen to the radio, read a book, or played |
| ¥ | Distraction | 3 | I went on the internet or used social media to distract myself |
| * | Social withdrawal | 4 | I stayed by myself |
| * | Social withdrawal | 5 | I kept quiet about the problem |
| * | Cognitive restructuring | 6 | I tried to see the good side of things |
| * | Self-criticism | 7 | I blamed myself for causing the problem |
| * | Blaming others | 8 | I blamed someone else for causing the problem |
| * | Problem solving | 9 | I tried to fix the problem by thinking of answers |
| * | Problem solving | 10 | I tried to fix the problem by doing something about it. |
| ¥ | Problem solving | 11 | I tried to fix the problem by talking to someone |
| * | Emotional regulation | 12 | I yelled, screamed, or got mad |
| * | Emotional regulation | 13 | I tried to calm myself down |
| * | Wishful thinking | 14 | I wished the problem had never happened |
| * | Wishful thinking | 15 | I wished I could make things different |
| * | Social support | 16 | I tried to feel better by spending time with others like family, grownups or a friend |
| * | Social withdrawal | 17 | I didn't do anything because the problem couldn't be fixed |
| ¥ | Emotional regulation | 18 | I prayed |
| ¥ | Social support | 19 | I went on the internet to get support |
| ¥ | Emotional regulation | 20 | I meditated |
| ¥ | Distraction | 21 | I did some kind of sport or physical activity |
| ¥ | Cognitive restructuring | 22 | I wrote down my thoughts (e.g. in a diary) |

*One of the 15 items in the original scale. Wording slightly modified after piloting or based on team suggestions.

 ¥ New items included for testing based on a review of the literature on coping, notably items emerging in participatory work in Carnegie School of Education, Leeds-Beckett University (2018:8), and team suggestions.

## Quantitative sample size and composition by school at each baseline and at endline

In Tanzania, the intervention was school-wide; participation was not confined to students who had taken part in the original baseline. For this reason, we administered a second baseline survey to students who had not taken the original survey but chose to join the intervention. Limited resources prevented us from conducting a full second baseline. Consequently, our sample consists of two baselines for two different populations (members of the original classrooms selected for the survey and other students who self-selected into the intervention).

In Vietnam, following implementation of the initial baseline survey, constraints imposed by the COVID-19 pandemic meant that the intervention took longer to start than anticipated. In the process, some students who had participated in the baseline had already graduated (students in the last grades of primary and secondary schools) or moved elsewhere. For these reasons, we administered a second baseline for every student in the intervention group (new participants, and those who had joined in the first baseline). The result is also two baselines for some intervention groups, and only one for the comparator group or new participants. The endline consists of all intervention students that remained part of the study (excluding dropouts), and as many of the same students included in the comparator group plus additional students to increase statistical power. The result is a sample of cross-sectional data and a subsample of panel data for both groups.

**Table S.2 – Sample composition in Vietnam and Tanzania by school**

|  | Baseline 1 | | Baseline 2 | | Endline | |
| --- | --- | --- | --- | --- | --- | --- |
|  | Comparator group | Intervention | Comparator group | Intervention | Comparator group | Intervention |
| Vietnam | 746 | 98 | - | 290 | 706 | 187 |
| Nha Trang |  |  |  |  |  |  |
| Urban – lower secondary (1) | 76 | 11 | - | 38 | 65 | 18 |
| Urban – lower secondary (2) | 116 | 3 | - | 32 | 112 | 13 |
| Urban – upper secondary (1) | 112 | 19 | - | 37 | 104 | 20 |
| Urban – upper secondary (2) | 80 | 4 | - | 39 | 78 | 15 |
| Vinh City |  |  |  |  |  |  |
| Urban – lower secondary | 98 | 29 | - | 36 | 89 | 30 |
| Rural – lower secondary | 52 | 29 | - | 36 | 52 | 29 |
| Urban – upper secondary | 92 | 0 | - | 34 | 68 | 27 |
| Peri-urban – upper secondary | 120 | 3 | - | 38 | 138 | 35 |
|  |  |  |  |  |  |  |
| Tanzania | 378 | 22 | 11 | 78 | 300 | 100 |
| Mwanza |  |  |  |  |  |  |
| Peri-urban – secondary | 81 | 19 | 0 | 3 | 78 | 22 |
| Rural – primary | 100 | 0 | 3 | 27 | 73 | 27 |
| Morogoro |  |  |  |  |  |  |
| Peri-urban – primary | 97 | 3 | 2 | 24 | 73 | 27 |
| Urban – secondary | 100 | 0 | 6 | 24 | 76 | 24 |

**Note:** In Tanzania, all interventions observations constitute panel data, while the comparator data is cross-sectional. By contrast, in Vietnam, only a subsample of the intervention group (187 students) and the comparator group (249 students) were followed longitudinally, forming panel data.

## Selection bias and response bias in the survey

We tested our sample for various forms of selection bias. A first potential source is demographic differences between the comparator and intervention groups. In Tanzania, baseline characteristics were largely comparable between the two groups; the only significant imbalance was in socioeconomic status. Intervention participants was much more likely to be in the high socioeconomic group (intervention = 45%, comparator = 26%, p=0.001) and less likely to be in the middle group (intervention = 38%, comparator = 62%, p=0.002). The sampling weights did not fully correct this imbalance.

In Vietnam, intervention participants were more likely to be girls (intervention = 63%, comparator = 46%, p < 0.001) and less likely to be from low socioeconomic status groups (intervention = 17%, comparator = 27%, p<0.001). Applying sample weights corrects for these demographic imbalances, the differences were no longer statistically significant.

A second source of bias, given respondent self-selection into the intervention, is potential differences in the mental health indicators between the comparator and intervention groups. In fact, for Tanzania, our comparison of baseline levels of our key mental health measures (MHLS, MHPK-10, Attitudes Toward Seeking Professional Psychological Help scale, SDQ mental health difficulties and prosocial behaviour subscales and KidCope Active Coping subscale) found the intervention group had higher scores across the board, sampling weights notwithstanding; the difference was statistically significant at the 95% level for all measures except the SDQ mental health difficulties subscale (*p*=0.265) and the Kidcope Active Coping subscale (*p*=0.361). However, in the regression analysis, after adjusting for sociodemographic differences and applying sampling weights, differences in mental health outcomes between comparator and intervention groups at baseline no longer predicted that outcome at endline, for all measures except the MHPK-10; in other words, the intervention effect appears robust to observed self-selection bias.

In the case of Vietnam, only a small but statistically significant differences was observed for MHLS after applying sample weights (intervention=.70, baseline=0.69, *p*=.03). For all other scales, differences between intervention and comparator at baseline level were not statistically significant.

Another limitation concerns questionnaire sensitivity and potential response bias. Although the study teams sought to ensure that students fully understood the survey questions, the answers received were accepted as final; there was no opportunity to probe or to clarify ambiguities, or to overcome missing responses, particularly for sensitive questions. The possibility of response bias arises because respondents had access to the full survey before responding to any single question, which might result in changing some of their responses to skip the follow-up questions. We found that including ‘I prefer not to say’ as a response to potentially sensitive questions increased the quality of the survey data, as significant numbers of students selected this option. In addition, it is possible that some students who agreed to participate in the survey at both baseline and endline might have responded without paying due attention, owing to exam fatigue and time constraints (at both baseline and endline, the surveys took place after the exam period).

## Details of ethics process

Ethical clearance was obtained from the ODI Research Ethics Committee (ref P000005), where the PI was based at the time. In Tanzania, ethical approval was obtained via the National Institute for Medical Research (NIMR). In Vietnam, permission was granted via the Provincial Department of Education and Training of the two cities where the study was implemented. Informed written consent was obtained from parents and/or teachers acting on behalf of parents, and from adolescents. The informed consent procedure included explaining any potential risks/benefits of participating, ensuring confidentiality and anonymity, explaining that participants could terminate their involvement in the survey at any time including once they had started and assuring participants that all data will be saved in a secure password protected site to which only the research team will have access.

## Existing literature on SBMH in Vietnam and Tanzania and details of the co-created process and intervention

**Existing SBMH interventions in Vietnam and Cambodia**

As mentioned in the manuscript, at the time of designing and implementing the AMP intervention, the study team was not aware of any similar projects, i.e. ones which were co-created and took a universal approach. However, in the past 2 years, there have been some publications on this theme which we briefly review here. Thus the universal school-based ‘Happy House’ (HH) intervention, adapted and translated from the Australian Resourceful Adolescent Program (RAP), was implemented in Hanoi (La NL et al. 2022). Findings from the evaluation showed the programme had positive and lasting (after 6 months) effects on psychological well-being, coping self-efficacy and anger management, in addition to reducing depressive symptoms (Tran et al. 2023). In Tanzania, while there have been some universal/whole school approaches, few focus on mental health, with the exception of (Berger et al. 2018; Kutcher et al. 2019; Kutcher et al. 2016), which respectively adopted the ERSAE-Stress-Prosocial (ESP) programme and a SBMH literacy programme from Canada – the African Guide (AG). Both programmes resulted in significant improvements in all outcome measures.

While these few universal SBMH programmes provide valuable insights into the effectiveness of such programmes in Tanzania and Vietnam, the current study advances this understanding. Critically, while the content of AMP is not dissimilar from the other programmes, it was co-created with adolescents as the end-users, but also parents and teachers, arguably making the interventions more relevant, and potentially also improving uptake and ultimately sustainability. Other key differences are also observed. The ESP and AG aim to train teachers to deliver the intervention; this was not the approach taken in AMP, though some teachers in Tanzania did support the facilitation. In addition, delivery modalities differ, e.g. HH ran 6 sessions on a weekly basis, AMP ran bi-monthly sessions over an 11-month period. Group sizes also differ - in HH there were between 40-45 students, in AMP ~30 – as do ages, with the EPS targeting primary school children (11-14), and AMPS, 10-19-year-olds.

**The AMP co-creation process**

Co-creation workshops lasting ~3 days were held in each school (4 in Tanzania, 8 in Vietnam). Participants were mostly adolescents – who then went on to participate in the intervention – but also some teachers and parents. In Tanzania the workshops were held in-person while in Vietnam because of COVID-19, most of the workshops – bar the final one where adolescents reviewed and finalized the prototype – were online. Across all schools and all participant types, 172 people took part in the workshops in Tanzania and 111 in Vietnam.

The full research team spent a few months designing together the co-creation workshops. These needed to be tailored to each context, also taking into account the research teams’ different levels of expertise in running these kinds of workshops. For instance, as mentioned by the research team, in a Vietnamese context, co-creation is not an easy concept for students to grasp as they are more accustomed to top-down approaches, where they listen rather than voice their opinions and perspectives (Myers and Samuels 2022). Hence, extensive discussions took place on how best to structure the co-creation workshops and encourage student engagement.

Although not published at the start of this study, the guidance provided by (Vargas et al. 2022)helps retrospectively structure the process that was used. Using their definition of co-creation, whereby “Co-creation refers to the collaborative approach of creative problem solving between diverse stakeholders at all stages of an initiative, from the problem identification and solution generation through to implementation and evaluation” pg1 (Vargas et al. 2022), a range of stakeholders were involved in the process (including adolescents, parents, teachers, local authority representatives) in all phases ranging from problem identification (through the baseline surveys) to solution design, implementation and evaluation, through regular check-ins and the endline data collection.

The resultant workshop agendas consisted of sessions where findings from the baseline study and literature review, conducted during the first phase of the project, were presented and used as scaffolding to inform the other sessions of the workshop. Thus, for instance, the baseline mixed method study – drawing on existing studies, including those that the research team had been involved in on related topics - asked students about drivers of mental ill-health as well as what was potentially protective; it also asked about their knowledge of what services existed to support them and what they would want services to look like. This was presented back to workshop participants, both to validate this but also to initiate thinking around the content of an intervention. The literature reviews identified various features of digital and non-digital mental health interventions that would be valuable to consider. For instance, a strong message coming out of the evidence (and confirmed also by the baseline findings, especially from the qualitative study (Samuels et al. 2022) was that blended approaches or ones that combine both in-person and online elements are more suitable for addressing youth mental health (Ananthakrishnan et al. 2020; Rost et al. 2020).

The sessions, which were delivered and facilitated by the Tanzania and Vietnamese research teams, thus combined presentations, small group work and plenary discussion. To help structure the small group work, a workbook was prepared which, again guided by the literature review and discussions with the country teams, outlined key decisions that the participants had to take to arrive at a draft prototype intervention. This included the content of the sessions, but also importantly how they would be delivered, by whom and how frequently. After the co-creation workshops, the research team completed the prototype, and those with expertise in digital tools designed this component. This was followed by sessions in which the research team shared the final versions with adolescents to ensure they remained faithful to their design (Myers and Samuels 2022).

**The co-created intervention**

In both countries, the initiative comprised hybrid digital and in-person elements (Samman et al. 2023; Samuels et al. 2023). In Tanzania, the ‘Psychology, Resilient Adolescent Minds’ (RAM) or ‘Happy Path’ clubs depending on the school and so named by the students, were facilitated by teachers (also referred to as mentors), who had taken part in both the baseline study and the co-creation workshops. These teachers also needed basic ICT skills to support in the digital component and prior to starting the intervention, they participated in a one-day refresher training encompassing facilitation skills, monitoring and supervision of club. They facilitated the in-person sessions which were held bi-monthly running over a 10-month period. These sessions combined discussions and debates, with sports and activities including games, writing poems and making posters. The sessions lasted between 1 to 2 hours and in each school the club had a chairperson, vice chairperson and secretary.

The digital component involved a computer-based learning platform containing a digital library with interactive mental health resources (including a moodtracker app in one school). The students used the school computers to take part in the digital component – in fact a key selection criteria for schools was that they had to have at least 10 functioning computer (as the project progressed, it was apparent that some schools computers needed upgrading and the project was able to find funds to support this (Kyungu 2023; Samman et al. 2023). Table 5.1 identifies the modules alongside the digital and in-person elements. Note that one module could last up to 3 sessions.

**Table 5.1 Overview of the co-created initiative in Tanzania**

| Module | **Digital** | **In-person** | **Session(s)** |
| --- | --- | --- | --- |
| 1 | Introduction to mental health | Football; rede & group discussion *(understanding mental health)* | 1 |
| 2 | Types of mental health disorders (anxiety, depression, PTSD) | Football; rede; breathing & grounding exercises; mindful walking meditation, word search puzzles *(anxiety symptoms & stress reduction)* | 3 |
| 3 | Drivers of mental ill-health and reduced psychosocial wellbeing | Football; rede, debate *(Is mental health more important than physical health?)* | 1 |
| 4 | Stigma, discrimination and mental illnesses | Football; rede, group discussion *(dealing with stigma),* debate *(Females are more likely to have a mental illness than males)* | 1 |
| 5 | Coping strategies and behaviour | Football; rede; problem-solving, poem/song creation *(promoting mental & emotional wellbeing)* | 2 |
| 6 | Treatment and care | Football; rede; group discussion *(sleep & mental health),* poster creation *(counselling relationship)* | 1 |
| 7 | Mental health service support and safeguarding | Football; rede, group discussion *(community mental health support systems, getting help scenarios)* | 1 |
| **Total** |  |  | **10** |

In Vietnam, the in-person portion of the intervention, or the ‘Psychology clubs’, so named by the students, also ran twice monthly, over an 11-month period. Sessions alternated between indoor and outdoor sessions, with a total of 20 sessions (11 indoor and 9 outdoor). The indoor sessions were facilitated by local psychologists, and the outdoor sessions, by both students and the local psychologists. Although it was originally intended that student leaders, who had also been part of the co-creation workshops, would facilitate all sessions, the workshop participants determined that expert facilitators were needed. Local psychologists, who are linked to the psychiatric hospitals at provincial level but also operate and conduct outreach beyond the provincial capitals, were hired on a part-time basis to help with the facilitation. The research team briefed these two psychologists (one per province) on the study and trained them on facilitation skills (Phuong et al. 2023; Samuels et al. 2023).

Table 5.2 below outlines the different topics covered in the indoor sessions. Each session lasted around 90 minutes and was held outside school hours. In the outdoor sessions, students learnt about mental health topics through activities such as traditional games (tug of war) or sports.

**Table 5.2 Indoor session topics**

| **Month** | **Topic** |
| --- | --- |
| February | Topic 1: Introduction |
| March | Topic 2: Overview about mental health |
| April | Topic 3: Stress |
| May | Topic 4: Anxiety |
| June | Topic 5: Depression |
| July | Topic 6: Emotional Regulation Skills |
| August | Topic 7: Conflicts and Social Relationships (1) |
| September | Topic 8: Conflicts and Social Relationships (2) |
| October | Topic 9: Bullying |
| November | Topic 10: Self-care and Promoting Well-being |
| December | Reflection |

In Vietnam, given the more widespread use of smart phones, the students proposed the use of a mobile application called MoodTracker along with a Facebook page. The app enabled students to record or rate their feelings (see Figure 5.1) and to keep a daily diary, with the app also sending reminders to the students to use it. The app also linked to mental health resources. The Facebook group – which was private and allowed students to post anonymously - was linked to the in-person sessions. It enabled students to post summaries of information from the Psychology Club sessions, other mental health information, and club photos, and enabled additional online conversations. The administrators and monitors of the groups included both students from the Psychology Club (on a rotational basis) and members of the research team (for further details of the findings around the digital component see Samuels et al. 2023.

**Figure 5.1 Moodtracker* recording / rating feelings**


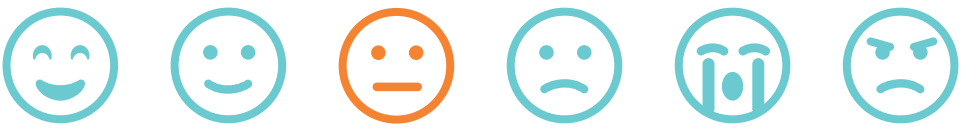


Each club in both countries had around 30 students, although attendance fluctuated by session according to, among other things, homework burdens. In both countries, research teams worked closely with the facilitators to prepare the sessions, also holding regular debriefing sessions after the intervention sessions. The outputs of this process were manuals in Vietnamese and Swahili containing detailed outlines of the sessions including the mental health content, but also the games and other activities that were suggested.

In both countries standardized templates tracked and monitored implementation for both in-person and digital activities. For the in-person component, the facilitators collected data on attendance; date/ time of the session; the theme (knowledge and activities); stakeholder participants; what worked well and less well; further suggestions; student feedback; photos and videos.

For the digital components, data was also collected monthly. In Vietnam data collected from the MoodTracker+ app included: login time, usage time, mood level (from 1 to 5) and monthly average mood level and any notes from students (why they chose that level, what happened, how they felt, etc.). The tracking of the Facebook group included recording of: time of posts, type of posts (photo, text or video) and number of photos and videos on each post; interactions (how many shares, views, comments) and reactions (how many like/love/laugh/sad/wow/angry). Similarly, in Tanzania data collected included: the average number of participants logged into each digital solution every month, the average number of times the digital solution was used monthly, the average mood in the app per month (through the mood tracker app), the average time each participant spent on the mood tracker monthly, and user comments regarding their overall experience with the digital solution.

The co-created interventions were not identical nor were they meant to be, as they were guided by the needs and priorities of the end-users/participants. Additionally, due to the overall adaptive nature of the project/study, following the tracking and monitoring process and two qualitative assessments in each country, some minor elements of the intervention were adjusted (e.g. the day of the week was changed in Tanzania).

## Number of survey respondents and their characteristics at baseline and endline, Tanzania and Vietnam

**Table 6.1 Profile of survey respondents, Tanzania (count and percentage of total)***

|  | **Baseline** | **Endline** |
| --- | --- | --- |
| **Total number of students** | 488 | 400 |
| **Gender** |  |  |
| Male | 247 (50.6) | 197 (49.25) |
| Female | 241 (49.4) | 203 (50.75) |
| **Age group** |  |  |
| 10-13 | 218 (44.7) | 165 (41.25) |
| 14+ | 270 (55.3) | 235 (58.75) |
| **SES Index** |  |  |
| Low SES | 68 (18.6) | 61 (15.25) |
| Middle SES | 165 (45.2) | 262 (65.5) |
| High SES | 132 (36.2) | 77 (19.25) |
| **Highest education of household head** |  |  |
| Some primary | 224 (60.5) | 148 (49.5) |
| Some secondary | 80 (21.6) | 74 (24.75) |
| More than secondary (technical,  university, etc.) | 66 (17.8) | 77 (25.75) |
| **Household composition** |  |  |
| Both mother and father | 236 (48.8) | 238 (59.5) |
| Only mother | 125 (25.8) | 76 (19.0) |
| Only father | 43 (8.9) | 25 (6.25) |
| Other | 80 (16.5) | 61 (15.25) |
| **Mother and father alive** |  |  |
| Both alive | 382 (78.9) | 357 (89.25) |
| Mother is alive | 65 (13.4) | 28 (7.0) |
| Father is alive | 23 (4.75) | 8 (2.0) |
| Neither is alive | 14 (2.9) | 7 (1.75) |
| **Religion** |  |  |
| Christian | 340 (70.1) | 277 (69.25) |
| Muslim | 143 (29.5) | 120 (30.0) |
| No religion / Other | 2 (.4) | 3 (0.75) |

*The numbers in each category do not sum to the total number of respondents owing to non-response. Each percentage is therefore the percentage of those responding to a given question.

**Table 6.2 Profile of survey respondents, Vietnam (count and percentage of total)***

*The numbers in each category do not sum to the total number of respondents owing to non-response. Each percentage is therefore the percentage of those responding to a given question.

## Qualitative themes and sub-themes

**Table 7.1 Qualitative themes and sub-themes**

| **Broad themes** | **Sub-themes** |
| --- | --- |
| Knowledge of mental health | - General knowledge of mental health / mental health conditions - Knowledge of specific mental health illnesses - Knowledge of drivers of mental ill-health |
| Knowledge of, and attitudes towards, mental health services and help-seeking behaviors | - Knowledge of mental health services - Knowledge of where to seek information - Attitudes toward mental health services and help-seeking behaviors (positive, negative/stigma) |
| Attitudes towards people with mental health problems | - Stigmatization and discrimination - Devalued, disdained, isolated - Neglected / ignored / indifferent |
| Perception of trends in prevalence of mental health issues over time | - Decrease in mental health issues - Little / no change |
| Drivers of mental ill-health | - Individual level – negative self-perception, lack of coping skills - Household level – family conflict, limited interaction between parents and children, high parental expectations - Beyond household - academic stress/pressure, conflicts with peers, peer pressure, peer victimization/bullying, stressful romantic relationships, technology - Gendered nature of drivers (girls more likely than boys to face mental ill-health - Particularly vulnerable populations (LGBTQI) |
| Protective factors for mental health | - Individual level – leisure, friendships, being cared for, needs met - Beyond household – doing well academically, Household level – strong family unit |
| Positive coping strategies | - Talking to/confiding in someone - Using distractions |
| Negative coping strategies | - Avoidant behaviors (e.g. sleeping, isolating), - Risky behaviors (e.g. emotional eating, self-harm, suicide ideation and substance abuse), - Aggressive behavior |

## Study phases / timeline


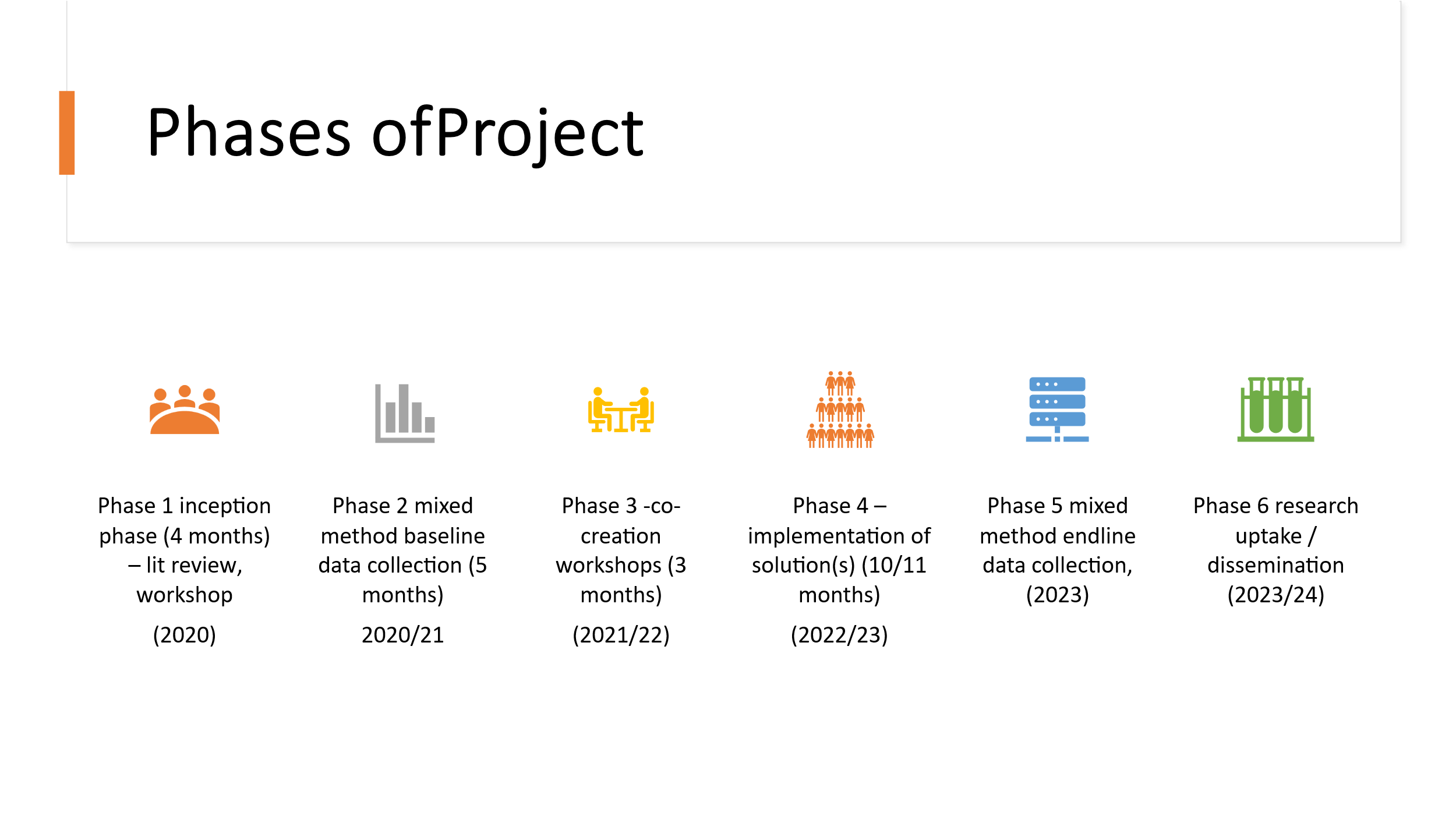


**References**

**Ananthakrishnan A, Samuels F, Marcus R and Leon-Himmelstine C** (2020) Non-digital interventions for adolescent mental health and psychosocial well-being: a review of the literature. London: ODI

**Berger R, Benatov J, Cuadros R, VanNattan J and Gelkopf M** (2018) Enhancing resiliency and promoting prosocial behavior among Tanzanian primary-school students: A school-based intervention. *Transcultural Psychiatry* **55**(6)**,** 821-845. <https://doi.org/10.1177/1363461518793749>.

**Bjørnsen HN, Eilertsen MEB, Ringdal R, Espnes GA and Moksnes UK** (2017) Positive mental health literacy: development and validation of a measure among Norwegian adolescents. *BMC Public Health* **17**(1)**,** 717. <https://doi.org/10.1186/s12889-017-4733-6>.

**Carnegie School of Education LBU** (2018) Mind your head – programme evaluation. UK: Leeds Beckett University

**Dow DE, Turner EL, Shayo AM, Mmbaga B, Cunningham CK and O'Donnell K** (2016) Evaluating mental health difficulties and associated outcomes among HIV-positive adolescents in Tanzania. *AIDS Care* **28**(7)**,** 825-833. <https://doi.org/10.1080/09540121.2016.1139043>.

**Elhai JD, Schweinle W and Anderson SM** (2008) Reliability and validity of the Attitudes Toward Seeking Professional Psychological Help Scale-Short Form. *PSYCHIATRY RESEARCH* **159**(3)**,** 320-329. <https://doi.org/https://doi.org/10.1016/j.psychres.2007.04.020>.

**Fischer EH and Farina A** (1995) Attitudes toward seeking professional psychologial help: A shortened form and considerations for research. *Journal of College Student Development* **36**(4)**,** 368-373.

**Goodman R, Ford T, Simmons H, Gatward R and Meltzer H** (2000) Using the Strengths and Difficulties Questionnaire (SDQ) to screen for child psychiatric disorders in a community sample. *Br J Psychiatry* **177,** 534-539. <https://doi.org/10.1192/bjp.177.6.534>.

**Hermenau K, Hecker T, Ruf M, Schauer E, Elbert T and Schauer M** (2011) Childhood adversity, mental ill-health and aggressive behavior in an African orphanage: Changes in response to trauma-focused therapy and the implementation of a new instructional system. *Child Adolesc Psychiatry Ment Health* **5,** 29. <https://doi.org/10.1186/1753-2000-5-29>.

**Hermenau K, Kaltenbach E, Mkinga G and Hecker T** (2015) Improving care quality and preventing maltreatment in institutional care - a feasibility study with caregivers. *FRONTIERS IN PSYCHOLOGY* **6**. <https://doi.org/ARTN> 937.10.3389/fpsyg.2015.00937.

**Hoosen N, Davids EL, de Vries PJ and Shung-King M** (2018) The Strengths and Difficulties Questionnaire (SDQ) in Africa: a scoping review of its application and validation. *CHILD AND ADOLESCENT PSYCHIATRY AND MENTAL HEALTH* **12**(1)**,** 6. <https://doi.org/10.1186/s13034-017-0212-1>.

**Kutcher S, Perkins K, Gilberds H, Udedi M, Ubuguyu O, Njau T, Chapota R and Hashish M** (2019) Creating Evidence-Based Youth Mental Health Policy in Sub-Saharan Africa: A Description of the Integrated Approach to Addressing the Issue of Youth Depression in Malawi and Tanzania. *Front Psychiatry* **10,** 542. <https://doi.org/10.3389/fpsyt.2019.00542>.

**Kutcher S, Wei Y, Gilberds H, Ubuguyu O, Njau T, Brown A, Sabuni N, Magimba A and Perkins K** (2016) A school mental health literacy curriculum resource training approach: effects on Tanzanian teachers’ mental health knowledge, stigma and help-seeking efficacy. *International Journal of Mental Health Systems* **10**(1)**,** 50. <https://doi.org/10.1186/s13033-016-0082-6>.

**Kyungu E** (2023) Co-creating, implementing and adapting mental health solutions for adolescents in schools in Tanzania – an overview. Milestone report. ODI.

**La NL, Shochet I, Tran T, Fisher J, Wurfl A, Nguyen N, Orr J, Stocker R and H N** (2022) Adaptation of a school-based mental health program for adolescents in Vietnam. *PLoS ONE* **17**(8).

**León-Himmelstine C, Samman E, Kyungu E, Roche J, Festo C, Plank G, Amani E, Samuels F and Pellini A** (2021)  Mental health and psychosocial well-being among adolescents in Tanzania: Findings from a mixed-methods baseline study. London: ODI.

**Myers C and Samuels F** (2022) ‘Let’s learn together’: co-creating mental health solutions with adolescents in Tanzania and Viet Nam. London: ODI.

**Nolan CP, O'Donnell PJM, Desderius BM, Mzombwe M, McNairy ML, Peck RN and Kingery JR** (2018) Depression screening in HIV-positive Tanzanian adults: comparing the PHQ-2, PHQ-9 and WHO-5 questionnaires. *Glob Ment Health (Camb)* **5,** e38. <https://doi.org/10.1017/gmh.2018.31>.

**Nyangara F OW, Kalungwa Z, Thurman TR** (2009) Community-Based Psychosocial Intervention for HIV-Affected Children and their Caregivers: Evaluation of The Salvation Army Available at <https://www.measureevaluation.org/resources/publications/sr-09-50.html> (accessed.

**O'Connor M and Casey L** (2015) The Mental Health Literacy Scale (MHLS): A new scale-based measure of mental health literacy. *Psychiatry Res* **229**(1-2)**,** 511-516. <https://doi.org/10.1016/j.psychres.2015.05.064>.

**Phuong NL, Minh DH, Van VH, Ha HT, Dao KTA, Ha LV, Myers C, Pellini A, Roche JM and Samuels F** (2023) Developing a School-Based Mental Health Promotion Program Through Co-creation Approach. *VNU Journal of Science: Education Research; Vol 39 No 3DO - 10.25073/2588-1159/vnuer.4752*.

**Powell TM, Wegmann KM and Overstreet S** (2019) Measuring adolescent coping styles following a natural disaster: An ESEM analysis of the Kidcope. *School Mental Health: A Multidisciplinary Research and Practice Journal* **11**(2)**,** 335-344. <https://doi.org/10.1007/s12310-018-9288-x>.

**Rost L, Samuels F, Leon-Himmelstine C and Marcus R** (2020) Digital approaches to adolescent mental health: A review of the literature. London: ODI.

**Samman E, Kyungu E, Mshiu J, Samuels F, Roche J, Amani E, León-Himmelstine C, Plank G, Chakraborty R and Pellini A** (2023) The co-creation and implementation of an adolescent school-based mental health intervention in Tanzania: key findings. London: ODI.

**Samuels F, Roche JM, Dang H-M, Ho H, Nguyen P, Vu V, Kieu D, Nguyen N, Le H, Samman E, Georgia Plank, Chakraborty R and Pellini A** (2023) The co-creation and implementation of an adolescent school-based mental health intervention in Viet Nam: Key findings. London: ODI.

**Samuels F, Roche JM, Dang H-M, Ho H, Vu V, Nguyen P, Nguyen N, Samman E, Georgia Plank and Pellini A** (2022) Mental health and psychosocial wellbeing among adolescents in Viet Nam: findings from a mixed-methods baseline study. ODI: London.

**Spirito A, Stark LJ and Williams C** (1988) Development of a brief coping checklist for use with pediatric populations. *J Pediatr Psychol* **13**(4)**,** 555-574. <https://doi.org/10.1093/jpepsy/13.4.555>.

**Topp CW, Østergaard SD, Søndergaard S and Bech P** (2015) The WHO-5 Well-Being Index: A Systematic Review of the Literature. *Psychotherapy and Psychosomatics* **84**(3)**,** 167-176. <https://doi.org/10.1159/000376585>.

**Tran TD, Nguyen H, Shochet I, Nguyen N, La N, Wurfl A, Orr J, Nguyen H, Stocker R and Fisher J** (2023) School-based universal mental health promotion intervention for adolescents in Vietnam: Two-arm, parallel, controlled trial. *Glob Ment Health (Camb)* **10,** e69. <https://doi.org/10.1017/gmh.2023.66>.

**Vargas C, Whelan J, Brimblecombe J and Allender S** (2022) Co-creation, co-design, co-production for public health – a perspective on definitions and distinctions. *Public Health Research & Practice* **32**(2 DOI - <http://dx.doi.org/10.17061/phrp3222211>)**,** e2022.

1. Reliability was assessed using Cronbach’s alpha coefficient, which measures the internal consistency of a scale or the extent to which the individual components are measuring the same underlying construct. It can range between 0 and 1, with higher values indicating greater reliability. Generally, coefficient values of 0.6 or higher are considered acceptable and values 0.7 or higher are considered ‘good’. [↑](#footnote-ref-1)
