# Supplementary material for: Promoting adolescent mental health in Tanzania and Vietnam through a co-created universal school-based initiative: Findings from a mixed method study
Source: Glob Ment Health (Camb). 2025 Dec 1;12:e142. doi: 10.1017/gmh.2025.10100 (PMC12720375; doi:10.1017/gmh.2025.10100)
Supplement: Peer review history

# Reviewer report: Review: Promoting adolescent mental health in Tanzania and Vietnam through a co-created universal school-based initiative: Findings from a mixed method study — R0/PR2

**DOI:** 10.1017/gmh.2025.10100.pr2

This study provides valuable insights into a co-created school-based mental health (SBMH) program implemented in Tanzania and Vietnam. The mixed-methods approach strengthens the robustness of the findings, and the research contributes to the growing body of knowledge on adolescent mental health interventions in low- and middle-income countries (LMICs). However, certain aspects require further clarification and elaboration to enhance reproducibility, statistical rigor, and the study’s novelty in the field.

Background

1. The introduction effectively highlights the mental health challenges faced by adolescents in LMICs and the need for school-based interventions. However, the theoretical foundation of the co-creation approach and its distinct advantages over existing SBMH models should be articulated more clearly.

2. The manuscript states that this is the first co-created SBMH initiative in Tanzania and Vietnam, but how does it build upon or differ from previous participatory mental health interventions? - Providing a clearer theoretical or conceptual framework would strengthen the study’s novelty.

3. The cultural and structural differences between Tanzania and Vietnam regarding mental health perception and education systems should be discussed in more detail to contextualize the study’s cross-country comparisons.

Methods

4. While the intervention design is well-described, more detailed information on the program’s structure, content, and delivery methods would improve reproducibility.

5. The role of facilitators (e.g., teachers, psychologists, peer mentors) and their training process should be described more explicitly. How was fidelity to the intervention ensured across schools and countries? Were there any standardized manuals or monitoring mechanisms?

6. The study would benefit from reporting effect sizes (Cohen’s d, partial η²) along with p-values to provide a clearer understanding of the practical significance of the intervention effects.

7. The potential clustering effect due to school-based intervention should be addressed. Since participants were nested within schools, employing hierarchical linear modeling (HLM) or generalized linear mixed models (GLMM) could account for intra-school correlations.

8. Given the number of outcome variables analyzed, multiple comparison corrections (e.g., Bonferroni or False Discovery Rate adjustments) should be considered to mitigate Type I error risks.

Results

9. The results demonstrate statistically significant improvements in mental health literacy (MHL) and help-seeking attitudes, particularly among certain subgroups (e.g., male students and lower SES participants). However, the magnitude of these changes should be contextualized. Are they clinically or educationally meaningful?

10. In Vietnam, emotional-focused coping strategies decreased, while active coping did not increase significantly. Could this be due to differences in intervention intensity, cultural differences in coping mechanisms, or program content? A deeper exploration of these potential explanations would be beneficial.

11. The observed gender differences in help-seeking attitudes and mental health outcomes are important, but further discussion on underlying mechanisms (e.g., cultural gender norms, stigma, school policies) would enhance the interpretation of findings.

12. The differences in intervention effectiveness between Tanzania and Vietnam should be discussed with greater nuance. Could they be attributed to intervention dose (bi-weekly vs. monthly), school environment, or pre-existing mental health literacy levels?

Discussion

13. The study presents important evidence for SBMH interventions in LMICs, but its novelty should be more explicitly articulated.

14. How does this research extend existing frameworks in participatory mental health interventions? What specific policy recommendations can be drawn for integrating similar programs into school curricula?

15. The study effectively demonstrates short-term benefits, but its long-term sustainability remains unclear.

16. Did the study include any follow-up assessments beyond the endline? If not, do the authors have plans for longitudinal data collection to examine whether the observed improvements persist over time?

17. How feasible is it to institutionalize the program within school systems? Are there any ongoing efforts to scale up the intervention through government or NGO collaborations?

18. While the discussion acknowledges some limitations, a more thorough examination of potential biases would strengthen the manuscript. For example:

- Self-report bias: Mental health outcomes were measured through self-reported surveys, which may be influenced by social desirability bias.

- Selection bias in qualitative data: Were students with negative experiences also included in qualitative interviews? How were participants selected for focus groups?

- External validity: To what extent can the findings be generalized to other LMICs with different sociocultural and educational contexts?

# Reviewer report: Review: Promoting adolescent mental health in Tanzania and Vietnam through a co-created universal school-based initiative: Findings from a mixed method study — R0/PR3

**DOI:** 10.1017/gmh.2025.10100.pr3

Overall, this study was not rigorously designed. The manuscript lacks many important details. I suggest dividing it into two separate manuscripts. For example, the authors could divide it by country, as the interventions in the two countries were not identical and the study designs were significantly different, making the results difficult to compare. Another option is to separate the quantitative and qualitative studies. The qualitative component includes a large amount of data, and if presented together with the quantitative results, the paper may become excessively lengthy. Additionally, the quantitative and qualitative components address different research questions and should be presented separately.

Abstract:

1. The study’s aims should be stated more clearly.

2. The description of the quantitative study design as “pre- and post-intervention panel and cross-sectional surveys” is difficult to understand.

3. The conclusion, “co-created SBMH promotion can significantly strengthen under-resourced systems in LMICs,” should be revised. This study does not provide data related to “strengthening the systems.”

Background:

4. The background should include a review of studies evaluating the effectiveness of school-based interventions on students’ mental health in Tanzania and Vietnam.

5. The research aims and objectives should be stated explicitly rather than the project objectives.

Methods:

6. The Methods section should include more subheadings—such as Study Design, Settings, Intervention, Quasi-Experimental Component, and Qualitative Component—to enhance readability. The Quasi-Experimental and Qualitative Component sections may also require subheadings like Participants, Measures/Interview Guide, Procedures, and Analysis.

7. It is unclear why mixed methods were used and which research questions were addressed by the quantitative and qualitative components.

8. Clarify what the “project team” did with the randomly sampled classrooms. How were participants selected?

9. Provide details about the consent process.

10. The authors stated, “The intervention took place at every school, with the comparator group consisting of non-participating youth in the same schools.” Does this mean the intervention and control groups could be in the same classes if some students in the selected classes refused to participate?

11. How did the study control for contamination when the intervention and control groups were in the same schools and even in the same classes?

12. The descriptions of the measures used in the survey should be expanded, including details on content, scoring, and local evidence of psychometric properties.

13. What potential confounders or covariates were assessed in this study and on what basis those were selected?

14. How were the analyses adjusted for clustering at the school and class levels?

15. This study had multiple outcomes. Clarify whether adjustments for multiple testing were applied. If so, specify the statistical methods used for these adjustments.

16. How were missing data handled? Report the proportions of missing data.

17. What factors were controlled for in the models for Tanzania?

18. The authors stated, “Where sample sizes permitted, gender, age, and socio-economic status were included as covariates, enabling a greater understanding of factors underlying the intervention effects.” The phrase “where sample sizes permitted” should be elaborated upon.

19. The description of the qualitative component’s methods and data analysis should be expanded significantly.

20. Provide a timeline specifying the dates of baseline surveys, “co-creation workshops,” intervention implementation, endline surveys, and qualitative data collection.

21. Elaborate on how the intervention was developed.

22. The authors stated, “In Tanzania, teachers or mentors facilitated the in-person portion with bi-weekly sessions (Table 1) running over a 10-month period.” This suggests more than 20 sessions, which is inconsistent with Table 1 (which lists only 10 sessions).

23. Table 1 appears to apply to Tanzania. Was the intervention format the same for Vietnam?

24. The Limitations section should be placed under the Discussion.

25. Baseline Surveys 1 and 2 were not described in the Methods section.

Results and Discussion:

25. Tables A3 and A4 should be divided by intervention and control groups.

26. The detailed outputs of the statistical models should be presented in tables.

27. The description of the results, such as “In Tanzania, participants registered gains of 16% on the MHLS (p=0.000) and 9.5% for the MHPK-10 (p=0.000), while gains for the comparator group were 6% for each scale (p=0.000),” is unclear. Specify what 16% refers to—was it the increase in the mean MHLS score or the change in the proportion of participants with “high” MHLS score?

28. The qualitative results are not presented appropriately. A large amount of data was collected, but only a small portion has been reported.

29. The Discussion section should be expanded to include comparisons with previous studies.

# Reviewer report: Review: Promoting adolescent mental health in Tanzania and Vietnam through a co-created universal school-based initiative: Findings from a mixed method study — R0/PR4

**DOI:** 10.1017/gmh.2025.10100.pr4

Thank you for the opportunity to review this timely and important paper. The authors present the findings of a universal, school-based mental health initiative that was co-created with adolescents in Tanzania and Vietnam and evaluated through a mixed-methods design. The study stands out for its relevance, given the current debates about universal school-based mental health programmes, especially in light of recent inconclusive findings from large-scale trials in high-income countries. This manuscript offers a counterpoint by showcasing how universal mental health programmes, when adapted to local contexts and shaped through youth involvement, may have a place in under-resourced school systems where the burden of untreated mental health difficulties is high. That said, I thought the authors underplayed the universal approach taken in their study. In fact, reference to universal approaches is only made twice in the manuscript, and then twice in the reference list. I was surprised that the authors had not positioned this paper more strongly within the universal mental health literature, and in response to the big debate about the future of these approaches in schools. The term “universal” is also absent from the title, abstract, and discussion, and the authors miss the opportunity to position their work more explicitly within these ongoing debates. The authors may wish to consult the series of debate pieces about this in the journal, child and adolescent mental health. Key to the debate FOR universal approaches, is that they are key in LMICs where large treatment gaps and stigma persist and several authors in their pieces strongly advocate that these approaches learn from the challenges faced by large trials like MYRIAD, and deliberately and carefully using either human centred design on PAR involve youth throughout. It would really strengthen this paper to include this in the introduction.

The study has many strengths. It brings together data from two LMICs, employs a hybrid digital and in-person delivery model, and integrates both quantitative and qualitative data sources to assess intervention outcomes. The effort to centre youth perspectives is a key strength and contribution to the literature.

Despite these strengths, there are a number of issues (mostly methodological) that need to be addressed.

1. It may be helpful to specify the type of mixed-methods design used in this study for example, convergent parallel, explanatory sequential, etc.

2. There seemed to be a substantial amount of qualitative data collected, yet only a fraction of these data are presented and used to supplement the quan findings, but also in a very limited way. Also, not much is captured in the section on data analysis regarding how the various sources of data were triangulated, interpreted and organised to present a coherent picture of the findings.

3. The authors used various qualitative methods including in-depth interviews, focus group discussions, key informant interviews, and intergenerational family case studies, yet the rationale for these specific methods and how they aligned with each of the study’s three objectives is unclear. As such, it was unclear what the purpose of each of these approaches was beyond just that different participants were included in each.

4. The co-creation process is highlighted as a central innovation of the study (and it is!), yet it is not entirely clear how far this process extended in practice. While three-day workshops were held with adolescents and others, and efforts were made to integrate their perspectives into the intervention design, the process appears structured and researcher-led, including the use of pre-prepared workbooks and final synthesis by the researchers. The extent to which adolescents had real decision-making authority or influence over key components of the initiative particularly in its delivery or evaluation is not fully articulated. As such, while the manuscript draws on the language of participatory action research, it does not present evidence of youth involvement in implementation, data analysis, or dissemination. I also couldn’t see that any young person/persons were included on this paper as co-authors. This raises some concerns about the potential gap between the participatory claims made and the actual power-sharing that occurred. Greater transparency is needed to clarify the scope and limits of youth involvement. Another example would be on the decision of outcome variables, did adolescents have a say in what is measured about them and how? Give input into measurement items, and interview/focus group schedules etc? Read this paper?

5. How exactly was randomisation of the classes done? There seemed to be a lot of selection bias in the study.

6. In the quantitative component, a battery of well-known measures were used. However, no information is provided on the psychometric properties of these measures in either setting. There is no discussion of translation procedures, cultural adaptation, internal consistency, or construct validity. Of particular concern is the use of the Kidcope scale, which was subdivided into three coping categories (active, avoidant, and emotional/expressive) without explanation or reference to a validated structure. The absence of exploratory factor analysis on this scale, for example, somewhat undermines the conclusions drawn from these subscales.

7. The authors report emotion focused coping decreased by 14.4%. This is positive but the confidence interval is very wide (for the other measures too), and the p-value seems incorrect (p = 0.32).

8. No effect sizes are reported, and there seemed to be no adjustment made for clustering or corrections made for multiple comparisons? No information on how missing data were treated. Not clear what kind of logistic regression was done.

9. The authors state that thematic analysis was used for the qual data, but do not specify the specific type of thematic analysis used or whether data were coded inductively or deductively. Other details are how many researchers were involved in coding? Development of a codebook? whether coding consistency was checked across sites? There is no mention of reflexivity, intercoder reliability or discussion, or data saturation/information power.

10. The discussion section is underdeveloped. Some detail is introduced for the first time such as the existence of “clubs” in Tanzanian schools and does not sufficiently connect the findings to current universal school-based mental health literature.

# Editor report: Recommendation: Promoting adolescent mental health in Tanzania and Vietnam through a co-created universal school-based initiative: Findings from a mixed method study — R0/PR5

**DOI:** 10.1017/gmh.2025.10100.pr5

**Affiliations:** Psychiatry, Makerere University CHS, Uganda

Dear Ms Samuels Fiona,

Your manuscript ‘ Promoting adolescent mental health in Tanzania and Vietnam through a co-created school-based initiative: findings from a mixed method study’, has now been reviewed,

# Editor report: Decision: Promoting adolescent mental health in Tanzania and Vietnam through a co-created universal school-based initiative: Findings from a mixed method study — R0/PR6

**DOI:** 10.1017/gmh.2025.10100.pr6

**Affiliations:** Johns Hopkins University Bloomberg School of Public Health, United States

No accompanying comment.

# Reviewer report: Review: Promoting adolescent mental health in Tanzania and Vietnam through a co-created universal school-based initiative: Findings from a mixed method study — R1/PR8

**DOI:** 10.1017/gmh.2025.10100.pr8

Thank you for the opportunity to review the revisions to this paper, which the authors have meticulously attended to. There are some typographical and sentence errors scattered throughout the paper, which need some attention. I also appreciated the supplementary materials and the rich information captured there. Just perhaps to check whether you have ethics permission or permission from the schools to publish the name of the schools? See table S2 for example. Some of the tables are unclear/not easy to read in the SM - may need to be redrawn e.g. 5.1. I would also recommend that the reporting of the stats be double checked by the authors. Finally, the description of the way in which the qualitative data were analyzed seems oversimplified, and also confusing. Combining both IPA and RTA just did not make sense here, and unclear how this was practically implemented in terms of the various steps associated with each of these qualitative analytic approaches.

# Editor report: Recommendation: Promoting adolescent mental health in Tanzania and Vietnam through a co-created universal school-based initiative: Findings from a mixed method study — R1/PR9

**DOI:** 10.1017/gmh.2025.10100.pr9

**Affiliations:** Psychiatry, Makerere University CHS, Uganda

Dear Ms Fiona Samuels,

Your revised manuscript: ‘Promoting adolescent mental health in Tanzania and Vietnam through a co-created universal school-based initiative: Findings from a mixed method study’, has now been revised

# Editor report: Decision: Promoting adolescent mental health in Tanzania and Vietnam through a co-created universal school-based initiative: Findings from a mixed method study — R1/PR10

**DOI:** 10.1017/gmh.2025.10100.pr10

**Affiliations:** Johns Hopkins University Bloomberg School of Public Health, United States

No accompanying comment.

# Editor report: Recommendation: Promoting adolescent mental health in Tanzania and Vietnam through a co-created universal school-based initiative: Findings from a mixed method study — R2/PR12

**DOI:** 10.1017/gmh.2025.10100.pr12

**Affiliations:** Psychiatry, Makerere University CHS, Uganda

Dear Dr Samuels,

Thank you for carrying out the minor revisions suggested by the reviewer,

# Editor report: Decision: Promoting adolescent mental health in Tanzania and Vietnam through a co-created universal school-based initiative: Findings from a mixed method study — R2/PR13

**DOI:** 10.1017/gmh.2025.10100.pr13

**Affiliations:** Johns Hopkins University Bloomberg School of Public Health, United States

No accompanying comment.
